# Design of Experiments Leads to Scalable Analgesic Near-Infrared Fluorescent Coconut Nanoemulsions

**DOI:** 10.3390/pharmaceutics17081010

**Published:** 2025-08-01

**Authors:** Amit Chandra Das, Gayathri Aparnasai Reddy, Shekh Md. Newaj, Smith Patel, Riddhi Vichare, Lu Liu, Jelena M. Janjic

**Affiliations:** School of Pharmacy, Graduate School of Pharmaceutical Sciences, Duquesne University, Pittsburgh, PA 15282, USA

**Keywords:** nanoemulsion, Quality by Design (QbD), celecoxib, coconut oil, macrophage, pain

## Abstract

**Background:** Pain is a complex phenomenon characterized by unpleasant experiences with profound heterogeneity influenced by biological, psychological, and social factors. According to the National Health Interview Survey, 50.2 million U.S. adults (20.5%) experience pain on most days, with the annual cost of prescription medication for pain reaching approximately USD 17.8 billion. Theranostic pain nanomedicine therefore emerges as an attractive analgesic strategy with the potential for increased efficacy, reduced side-effects, and treatment personalization. Theranostic nanomedicine combines drug delivery and diagnostic features, allowing for real-time monitoring of analgesic efficacy in vivo using molecular imaging. However, clinical translation of these nanomedicines are challenging due to complex manufacturing methodologies, lack of standardized quality control, and potentially high costs. Quality by Design (QbD) can navigate these challenges and lead to the development of an optimal pain nanomedicine. Our lab previously reported a macrophage-targeted perfluorocarbon nanoemulsion (PFC NE) that demonstrated analgesic efficacy across multiple rodent pain models in both sexes. Here, we report PFC-free, biphasic nanoemulsions formulated with a biocompatible and non-immunogenic plant-based coconut oil loaded with a COX-2 inhibitor and a clinical-grade, indocyanine green (ICG) near-infrared fluorescent (NIRF) dye for parenteral theranostic analgesic nanomedicine. **Methods:** Critical process parameters and material attributes were identified through the FMECA (Failure, Modes, Effects, and Criticality Analysis) method and optimized using a 3 × 2 full-factorial design of experiments. We investigated the impact of the oil-to-surfactant ratio (*w*/*w*) with three different surfactant systems on the colloidal properties of NE. Small-scale (100 mL) batches were manufactured using sonication and microfluidization, and the final formulation was scaled up to 500 mL with microfluidization. The colloidal stability of NE was assessed using dynamic light scattering (DLS) and drug quantification was conducted through reverse-phase HPLC. An in vitro drug release study was conducted using the dialysis bag method, accompanied by HPLC quantification. The formulation was further evaluated for cell viability, cellular uptake, and COX-2 inhibition in the RAW 264.7 macrophage cell line. **Results:** Nanoemulsion droplet size increased with a higher oil-to-surfactant ratio (*w*/*w*) but was no significant impact by the type of surfactant system used. Thermal cycling and serum stability studies confirmed NE colloidal stability upon exposure to high and low temperatures and biological fluids. We also demonstrated the necessity of a solubilizer for long-term fluorescence stability of ICG. The nanoemulsion showed no cellular toxicity and effectively inhibited PGE2 in activated macrophages. **Conclusions:** To our knowledge, this is the first instance of a celecoxib-loaded theranostic platform developed using a plant-derived hydrocarbon oil, applying the QbD approach that demonstrated COX-2 inhibition.

## 1. Introduction

According to the National Health Interview Survey, 50.2 million U.S. adults (20.5%) report pain on most days [1], and the annual cost of prescription medication for pain is around USD 17.8 billion [2]. National Ambulatory Medical Care Survey data show that around 36.4% of chronic pain patients receive opioids by prescription [3]. Opioid overdose during pain management substantially increases the mortality rate, and in 2021 alone, the National Center for Health Statistics reported around 80,000 deaths from opioid overdose [4]. The substantial heterogeneity, stemming from biological, psychological, and social factors, profoundly affects pain outcomes and response to treatment [5]. There is a profound need for safe, effective, and personalized pain treatments across healthcare, from post-surgical pain to chronic pain that arises from inflammatory diseases such as rheumatoid arthritis.

Nanomedicine offers a new path toward improving pain treatment efficacy, improved safety, and personalization, which may reduce the societal and economic burden of untreated pain. Nanomedicine, as a theranostic platform combining both therapeutic and imaging modalities [6], can be configured as an individualized treatment regimen. However, success in the development of quality nanomedicine is challenging due to hurdles such as safety in terms of biocompatibility, minimizing cytotoxicity and immunogenic response, adequate characterization, stability, and associated cost [7]. Moreover, the lack of product and process understanding impacts final product quality, and possess difficulties in reproducibility and scale-up. The Quality by Design (QbD) approach can guide the development of quality nanomedicine, cruising past the challenges. According to ICH Q8(R2), QbD is a systematic risk-based approach that initiates with a predefined objective and emphasizes product and process control [8]. QbD revolves around four key elements, starting with defining the target quality profile of the product [9], identification of the critical quality attributes (CQAs) [10], screening parameters that affect the CQAs, and lastly, estimating the role and impact of the parameters on quality attributes [11]. Risk assessment based on trial experiments, research articles, and prior experience helps to identify the factors that critically impact final product quality. The design of experiments (DoEs) is set up, using the factors to generate a design space suitable for manufacturing in a reproducible manner.

Our lab has already reported QbD-driven manufacturing of pain nanomedicine, where the therapeutic target is the macrophage [7,12,13]. Macrophages are the key immune cells for inducing inflammatory conditions in pain [12]. They induce inflammation by causing an imbalance in mediators like chemokines or cytokines [14]. In the earlier stage of inflammation, macrophages produce cytokines (IL-6, TNF-alpha), promote angiogenesis (generating new blood vessels to deliver nutrients and oxygen), and activate other types of immune cells like T-cells or NK cells [15]. However, persistent inflammatory stimulation causes overexpression of COX-2, resulting in chronic inflammation and further immune dysregulation by impacting T-cell function [16]. COX-2 also overexpresses the mPG2ES-1 (Microsomal prostaglandin E Synthase-1) enzyme, subsequently increasing PGE2 production. It is the ultimate key mediator for the inflammatory process [17]. Thus, inhibition of COX-2 decreases the production of PGE2, promoting relief from inflammation and pain. Celecoxib, a selective NSAID (nonsteroidal anti-inflammatory drug), belongs to the BCS class II drug with poor aqueous solubility, which was approved by the USFDA for the management of pain [18]. Celecoxib, when taken orally, damages the mucous layer of the stomach and causes ulceration and gastrointestinal bleeding [19]. Oil-in-water nanoemulsion can solubilize lipophilic drugs in its oil core and enhance systemic drug delivery. Nanoemulsions (NEs) are a kinetically stable but thermodynamically unstable form of colloidal dispersions with droplet diameters lower than 500 nm. The oil phase remains surrounded by amphiphilic surfactant molecules, lowering the interfacial energy and causing droplet formation [20]. Due to their high surface-area-to-volume ratio, nanoemulsions provide high drug entrapment efficiency with the potential for extended release [21] and improved bioavailability of poorly water-soluble drugs [22]. Nanoemulsions, prepared from plant-derived oils such as castor oil, olive oil, soybean oil, and palm oil, have already been reported to show analgesic and anti-inflammatory activity [23,24,25,26]. Our lab has previously shown that celecoxib-loaded perfluorocarbon nanoemulsion provides 4 days of pain relief with just a single dose of celecoxib (0.24 mg/kg) in a chronic constriction injury rat pain model [27]. In these studies, perfluorocarbons served as the imaging component of the theranostic nanoemulsions. Specifically, perfluorocarbons can be detected and imaged using fluorine-19 magnetic resonance imaging (19F MRI) in vivo and ex vivo [27,28].

Although being chemically inert and useful in preclinical studies to monitor the presence of nanoemulsions in target tissues and organs, perfluorocarbons are highly expensive, and the organ retention time can be more than 130 days [29]. The higher cost and retention in the body associated with perfluorocarbon is a major concern for the clinical translation of analgesic theranostic nanoemulsions. Thus, this article focuses on formulating a biphasic nanoemulsion, excluding the perfluorocarbon core but with similar colloidal stability and pharmacological effects. The purpose of this work is to develop a novel theranostic nanoemulsion that is safe, effective, and clinically viable as an analgesic. We used coconut oil, a plant-derived hydrocarbon oil, used in both the food and cosmetic industries. Coconut oil is a medium-chain oil consisting of saturated fatty acids, mostly lauric acid (45–53%) [30], and shows anti-inflammatory [31] and thermal resistance [32] properties. Furthermore, coconut oil has low viscosity because of the low intermolecular interaction among lauric acid molecules present in coconut oil. This facilitates the disruption of oil droplets at lower energy, leading to reduced droplet size [33]. Coconut oil nanoemulsion has already been reported to incorporate statin, resveratrol, and metformin for the treatment of burns, Alzheimer’s disease, and cancer, respectively [31,34,35]. The near-infrared fluorescent (NIRF) dye, indocyanine green (ICG), serves as the imaging moiety in this study, which enables the coconut oil celecoxib nanoemulsion to perform as a theranostic platform. However, thermal and aqueous instability limit the use of ICG. To overcome instability issues, conjugation with cationic lipid stearylamine (SA) [36] and encapsulation in solid lipid nanoparticles [37] are employed. The impact of surfactant and oil concentration on droplet size and drug encapsulation properties of the system is supported by evidence from several studies [38,39]. Another major focus of this study was to investigate the impact of the surfactant system on the formation and stability of nanoemulsions. However, the surfactant system blends were chosen to keep the HLB (hydrophilic–lipophilic balance) value in a narrow range (13.5–16), as changing the HLB value has distinct impacts on emulsion stability [40]. This investigation also focuses on the impact of the surfactant system and oil-to-surfactant ratio (O/S ratio) on the colloidal properties and encapsulation efficiency of nanoemulsion. The results from the experimental design statistically proved that the O/S ratio (*w*/*w*) has a significant impact on droplet size reduction. The presented bi-phasic analgesic platform also retained similar colloidal and encapsulation properties when scaled up to five times the volume using a different manufacturing approach and equipment. Collectively, in this study, we developed a celecoxib-loaded nanoemulsion containing NIRF dye in the hydrocarbon oil core, applying the QbD approach. This theranostic platform showed stable scaling-up capacity, exhibiting COX-2 inhibition in vitro.

## 2. Materials and Methods

### 2.1. Materials

All the chemicals were purchased from commercially available sources and were used as is. The surfactants (Pluronic^®^ P105, Pluronic^®^ F127 (Poloxamer 407), Pluronic^®^ P123 (Poloxamer 403), and Cremophor^®^ EL (polyethoxylated castor oil)) were purchased from Sigma-Aldrich (St. Louis, MO, USA), Spectrum (New Brunswick, NJ, USA), and US Biological Life Sciences (Salem, MA, USA). Coconut oil was purchased from MP Biomedicals, Miglyol^®^ 812N was purchased from CREMER Oleo Product Division (Hamburg, Germany), Transcutol^®^ (2-(2-ethoxyethoxy)-ethanol) was purchased from Spectrum (New Brunswick, NJ, USA), and indocyanine green (ICG) dye was purchased from Sigma-Aldrich (St. Louis, MO, USA). Celecoxib was purchased from LC Laboratories (Woburn, MA, USA). Sonicator from Fisher Scientific (Waltham, MA, USA), Microfluidizer M110S, and Microfluidizer M110P from Microfluidics Corporation (Westwood, MA, USA) were used for nanoemulsion preparation.

### 2.2. Methods

Screening of formulation and process parameters was performed during the initial trial experiments to finalize the formulation materials and critical process parameters. During the trial experiment, the impact of microfluidization pass and pressure, sonication time, and incorporation of Miglyol in the formulation was assessed. Based on the results obtained from the trials, a comprehensive risk assessment was performed applying Failure Modes, Effects, and Criticality Analysis (FMECA).

#### 2.2.1. Risk Assessment and Experimental Design

Each unit operation of the manufacturing and formulation is considered one by one to check all sources of variability in the critical quality attributes of nanoemulsion. Each variability is considered a failure mode and calculated by assigning the risk priority numbers [9,41].

RPN = Severity × Frequency of occurrence × Detectability

Each failure mode obtained scores for severity, frequency of occurrence, and detectability on a scale of 1 to 5. 

We considered two formulation variables. The surfactant system varied at two levels, and the oil-to-surfactant ratio (O/S ratio) varied at three levels in a full factorial design with two center points, giving a total of 8 runs. JMP Pro 15 software was used to create the design of the experiment. A full factorial design enabled us to study all the possible main effects, and center points will allow us to investigate whether there is any interaction between the factors. 

#### 2.2.2. Micelle and Nanoemulsion Preparation

To prepare the micelle from a binary mixture of surfactants, the surfactants were dissolved separately in water by magnetic stirring at 350 rpm. After complete dissolving, the two surfactant solutions were combined and stirred well in a beaker. After complete dissolving of the surfactant mixture, water was added to bring the micelle to the desired final volume. The hydrophilic–lipophilic balance (HLB) value of the surfactant blend was calculated based on the following equation:

HLB_mix_ = F_S1_ × HLB_S1_ + F_S2_ × HLB_S2_

where HLB_mix_, HLB_S1,_ and HLB_S2_ are the HLB values of the surfactant mixture, surfactant 1, and surfactant 2, respectively, and F_S1_ and F_S2_ are the weight fractions of surfactant 1 and surfactant 2.

For small-scale (100 mL) batches, pre-weighed coconut oil was melted using a hot water bath at around 35–45 °C. Micelle was also kept in the hot water bath to maintain the same temperature as the oil phase. Celecoxib was then dissolved in coconut oil by magnetic stirring at 350 rpm for 1 h. The micelle was then added into the oil phase and stirred using the magnetic stirrer at 350 rpm, maintaining the temperature at 35–45 °C for 15 min. The pre-emulsion was kept in pre-heated water and sonicated for 4 min using the Sonicator at 39% amplitude and pulse for 30 s, with a 15 s pause in between. The final emulsification was completed using Microfluidizer M110S at 80 psi air pressure.

In the large-scale batches (500 mL), we dissolved the NIRF dye in the hydrocarbon oil phase along with celecoxib. Later, to achieve long-term fluorescence stability, we used Transcutol to dissolve the dye before adding hydrocarbon oil. Pre-emulsification was performed with a hand mixer for 15 s. The pre-emulsion was then subjected to microfluidization in M110P for two passes. The manufacturing approach for drug-free nanoemulsion (DF NE) was similar to that of celecoxib-loaded nanoemulsion (CXB NE) except for the addition of the drug.

#### 2.2.3. Colloidal, Fluorescence, and Morphological Analysis

Dynamic light scattering (Zetasizer Nano ZS, Malvern Instruments, Worcestershire, UK) was used to analyze the droplet diameter and polydispersity index (PDI). All the measurements were taken at room temperature (25 °C), and the nanoemulsion was diluted at a 1:40 dilution in DI water. For near-infrared (NIRF) fluorescence imaging of nanoemulsion, Li-Cor Odyssey M was used. The nanoemulsion was diluted in deionized water at an ICG concentration range of 5 μM to 0.156 μM. A measure of 100 μL of the dilution was transferred to a clear 96-well plate, and the fluorescence intensity was measured at 800 nm wavelength, keeping a 3.75 focus offset.

The morphology of CXB NE was studied using transmission electron microscopy (FEI Tecnai F20, Hillsboro, OR, USA). Copper grid activation was performed using a plasma cleaner for three seconds before the experiment. The grid was stained with CXB NE for one minute, and filter paper was used to remove any excess NE. A phosphotungstic acid (0.5% *w*/*v*, pH adjusted to 7) solution was used for staining the grid for 15 s before analyzing through TEM [42].

#### 2.2.4. Stability Study Under Stress Conditions

To investigate nanoemulsion stability upon exposure to biological fluid, nanoemulsions were diluted in 1.5 mL Eppendorf tubes at 1:40 *v*/*v* in three conditions: (a) DI water, (b) DMEM (Dulbecco’s Modified Eagle Medium), and (c) 20% FBS (fetal bovine serum) in DMEM. The dilutions were made in duplicate, and the first set of dilutions was immediately measured with DLS, which is the 0-h time point. The second set of dilutions was incubated at 37 °C for 72 h and then measured with DLS, which was the 72-h time point [43]. To check whether the nanoemulsions retain their colloidal properties under extreme temperatures, 2 mL of nanoemulsion was aliquoted from three different places in the container into the Eppendorf tube. The Eppendorf tubes were parafilmed and kept at 4 °C for 24 h. After 24 h, the tubes were transferred to a 50 °C incubator for 24 h. This cycle between 4 °C and 50 °C was repeated for four thermal cycles (8 days). After four cycles between 4 °C and 50 °C (8 days) were completed, the size and polydispersity index (PDI) were measured with DLS [43].

#### 2.2.5. Celecoxib Quantification and In Vitro Release Study

Celecoxib was quantified using HPLC (DIONEX Ultimate 3000, Thermo Scientific™, Waltham, MA, USA). A reverse-phase HPLC column (C18 Hypersil Gold 150 × 4.6 mm—Thermo Scientific) was used. For the calibration curve, celecoxib was dissolved in (75:25) methanol and water at different concentrations ranging from 10 μg/mL to 50 μg/mL. The sample was quantified using the same mobile phase of methanol and water at 30 °C, using a flow rate of 1 mL/min at a wavelength of 255 nm. The total run time was 6 min with the detection of celecoxib at a retention time of approximately 3.7 min. To quantify nanoemulsion encapsulation efficiency, the nanoemulsion was first dissolved in methanol in a 10 mL volumetric flask. The volumetric flask was then heated in a water bath to melt the coconut oil, as the addition of methanol solidified the oil. The samples were then placed into an Eppendorf tube and centrifuged; then, the supernatant was transferred to the HPLC vial for quantification.

An in vitro drug release study was set up following previously published articles from our group [12,44]. The dialysis bags (Thermo Fisher Scientific snakeskin dialysis tubing REF 68035) were cut and tied at both ends with thread after filling with CXB NE and free CXB drug solution. Each of the dialysis bags (triplicate, *n* = 3) was put inside a 50 mL Falcon tube containing 15 mL of release media (phosphate buffered saline with 25% methanol). The falcon tubes containing the dialysis bag were kept in a shaking incubator at 100 rpm, maintaining a temperature of 37 °C. As celecoxib solubility in methanol is very high (113.94 mg/mL) [45], methanol was added to the release media to maintain the sink condition [12]. Dialysis bags were transferred to a fresh Falcon tube with 15 mL of release medium at each time point (hours 2, 4, 6, 8, 24, 48, 72, 96, and 120). To evaluate the drug release pattern from CXB NE, the cumulative percentage of released drug was plotted against time. 

#### 2.2.6. In Vitro Cell Viability and Cellular Uptake Study

Cell viability upon exposure to nanoemulsion (CXB NE and DF NE) was assessed using the RAW 264.7 macrophage cell line (ATCC TIB 71, ATCC, Manassas, VA, USA) following previously published protocols [44,46,47]. Cells were plated at 10,000 cells per well in 96-well plates and incubated at 37 °C with 5% CO_2_ for 24 h. Cells in the 96-well plates were then treated with nanoemulsion in the cell culture medium from 0 to 80 μL (*n* = 6) for 20 h. Free celecoxib dissolved in dimethyl sulfoxide (DMSO), matching the concentration of CXB NE, was used as a free drug control. Viability was assessed using the CellTiter-Glo 2.0^®^ assay kit (Promega, Madison, WI, USA). The medium was removed after 24 h of treatment addition and replaced with 100 µL of fresh warm cell culture media. A measure of 40 µL of CellTiter-Glo reagent was added, and the plate was shaken at 50 rpm for 15 min. Cell lysates were transferred from the culture plate to an opaque white 96-well plate and run for Luminescence (BioTek Synergy HTX Multi-Mode reader, Agilent, Santa Clara, CA, USA).

For the cellular uptake study, RAW 264.7 (ATCC TIB-71) macrophage cells were plated in a chamber slide (TM system Lab-Tek@II, Thermo Fisher Scientific Inc., Waltham, MA, USA) at 20k cells/well in 0.5 mL full cell culture medium in each chamber. The chamber slides were then incubated at 37 °C with 5% CO_2_ overnight for attachment. The cells were treated with CXB NE (μL/mL) for 6 h. The exposed medium was washed away with 0.5 mL of warm 1X PBS, and the cells were fixed with 4% paraformaldehyde in 1X PBS solution (*w*/*v*) for 20 min. Cells were washed with 0.5 mL 1X PBS twice after fixation. A total of 3–4 drops of ProLong Diamond mountant were applied to the chamber slide after the chamber wall was removed. A cover slip was placed on the slide, which was then stored in a dark area for 2–3 h for DAPI nuclei staining. Cellular uptake images were taken with the Keyence microscope.

#### 2.2.7. In Vitro Pharmacological Response

The in vitro assessment of PGE2 inhibition was performed with independent cell culture plates (*n* = 3). Raw 264.7 macrophage cells were seeded in 6-well plates with 300,000 cells in each well/2 mL 10% FBS in DMEM. Moreover, 6-well plates were incubated at 37 °C and 5% CO_2_ for 48 h before exposure to CXB NE, DF-NE, free drug CXB, and free drug vehicle, dimethyl sulfoxide (DMSO), for 24 h. The actual celecoxib concentrations in CXB NE are 20 μM. Free drug CXB was dissolved in DMSO (10 mM) to match the CXB NE treatment concentration. To activate the macrophages, LPS in 10% FBS in DMEM (500 ng/mL) was added after removing the treatment culture medium for 18 h. After the cell culture supernatant was collected, it was spun down at 4 °C and 1100 rpm for 5 min. We used the spun supernatant to develop a Prostaglandin E2 ELISA kit -Monoclonal Cayman Chemical (No. 514010) at a wavelength between 405 and 420 nm. The ELISA was performed with a BioTek plate reader.

#### 2.2.8. Statistical Analysis

The data were analyzed using GraphPad Prism v10. The difference between the compared groups for droplet size analysis was performed with a two-tailed unpaired *t*-test, and for the drug release study, with two-way repeated measures analysis of variance (ANOVA). The statistical test details are also described in the legends in each figure, with the significance level set at *p* < 0.05.

## 3. Results

The primary purpose of this work is to develop the framework for manufacturing oil-in-water nanoemulsion with a complete understanding of the formulation and process impact on the final product. Adaptation of the Quality by Design (QbD) approach facilitates this understanding and the establishment of a transferable and scalable process to manufacture celecoxib-loaded nanoemulsion for effective pain relief. The quality target product profiles (QTPPs) were defined, comprising all the plausible attributes of the final product. This includes the dosage form, route of administration, dose, and colloidal attributes.

### 3.1. Identification of Critical Quality Attributes (CQAs)

After setting up the QTPPs (Table 1), all the possible attributes were critically analyzed and justified before selecting CQAs. The CQAs are stated with their target value in Table 2. The CQAs were selected considering the most critical attributes for ensuring product quality as a safe and effective drug delivery platform. The nanoemulsion droplet diameter on day 7 after manufacturing was targeted between 100 and 160 nm for effective macrophage uptake by phagocytosis [42]. A polydispersity index (PDI) < 0.2 would be required to maintain a narrow size distribution, as a wide distribution leads to destabilization through Ostwald ripening [48]. Less than 10% change in droplet diameter and PDI < 0.1 after 1 week of storage would indicate stability in that storage condition. Encapsulation efficiency is directly related to therapeutic efficacy and safety, and more than 80% of encapsulation efficiency is the target to achieve. Less than 10% deviation in droplet diameter was expected after the thermal cycling study, which would give us product stability in harsh environments (4 °C and 50 °C). A change in size after serum stability will be checked only on the final selected formulation. A less than 20 nm change in droplet diameter in the presence of electrolytes and protein would indicate nanoemulsion stability, which is relevant as the nanoemulsion is intended for parenteral application.

### 3.2. Manufacturing Feasibility, Formulation Compatibility, and Process Parameter Screening

As there is no precedent for manufacturing coconut oil nanoemulsion using the high-energy emulsification method (microfluidization), the feasibility of the manufacturing approach needs to be studied.

**Figure 1:**
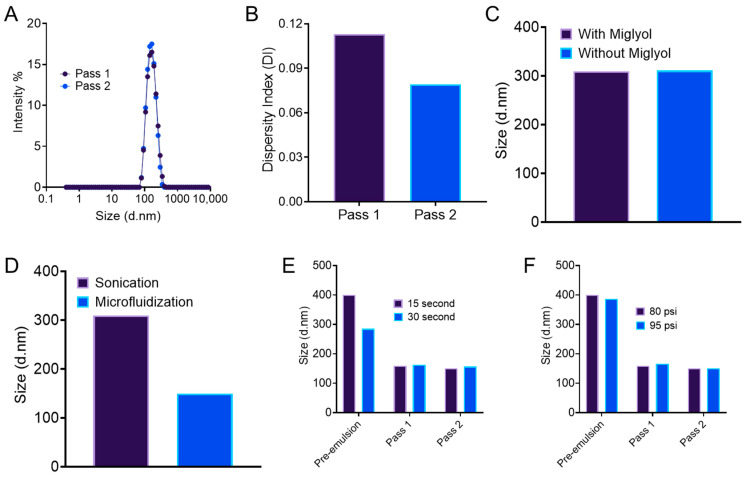
Assessment of manufacturing possibility, formulation compatibility, and process parameters. (**A**) Droplet size distribution. (**B**) Polydispersity index (PDI) of large-scale (500 mL) trial in M110P microfluidizer with coconut oil and F127/CrEl surfactant system. (**C**) Comparative droplet size of two batches of nanoemulsion with and without solubilizer (Miglyol 812). (**D**) Comparative droplet size of two batches of nanoemulsion manufactured with sonication and microfluidization only. Comparative droplet size of two batches of nanoemulsion processed at (**E**) 80 psi and 90 psi processing pressure and (**F**) 15 s and 30 s of sonication. All data in Figure 1 are from the manufacturing trials during screening and are given as *n* = 1.

To assess the manufacturing feasibility by microfluidization, we started with a 500 mL batch nanoemulsion. However, during microfluidization, we iced the machine before using it. This leads to the solidification of coconut oil inside the microfluidizer and the loss of nanoemulsion volume. This suggests that during microfluidization, the temperature needs to be maintained above the melting point of coconut oil (24–25 °C) [49]. Figure 1A,B showed that for two passes in microfluidization, though the droplet size distribution remains similar, the polydispersity index of the nanoemulsion was decreased below 0.1. This result suggested incorporating two passes in the future microfluidization steps. The comparison between two batches of nanoemulsion formulated with and without Miglyol, a co-solubilizer, did not show any difference in droplet size (Figure 1C). Figure 1D shows the necessity of microfluidization for droplet size reduction compared to sonication. During the manufacturing of small-scale batches, we employed sonication for pre-emulsification and microfluidization as the final processing step. Figure 1E,F show the screening of processing parameters such as microfluidization pressure and sonication time. Though microfluidization pressure between 80 and 90 psi did not show any difference in droplet size reduction, a sonication time of 30 s resulted in a size change of 100 nm in pre-emulsion.

### 3.3. Risk Assessment and High-Risk Factor Distribution

**Figure 2:**
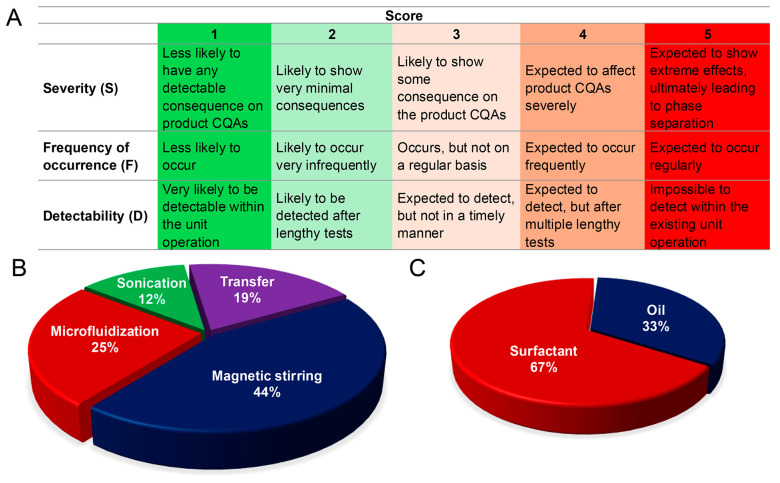
Risk assessment. (**A**) Scoring the risk factors for severity, frequency of occurrence, and detectability. High-risk factor distribution in (**B**) unit operation and (**C**) formulation.

Based on the trials and knowledge from the literature, the risk priority numbers were assigned to each potential risk factor belonging to all the unit operations, as well as the formulation. The RPN scoring matrix is shown in Figure 2A. Depending on the RPN value, risk factors were classified into low-, medium-, and high-risk categories. RPN values lower than 15 are considered low-risk, between 15 to 40 are considered medium-risk, and above 40 are considered high-risk factors. Abridged Failure Modes, Effects, and Criticality Analysis (FMECA) with the cause of failure and risk sources is listed in Table S1. Figure 2B shows the distribution of high-risk factors in all the processing unit operations. Several failure modes, such as improper mixing of oil and aqueous phase, lowering of temperature below the melting point of coconut oil, and incomplete dissolving of celecoxib in the oil phase, all happen during the magnetic stirring steps. This corresponds to 44% of the high-risk factor distribution in magnetic stirring, making it one of the critical sources of product failure. Although the change in temperature poses a higher risk, in this study, rather than studying the impact of temperature change on nanoemulsion, we tried to maintain the temperature just above the melting point of coconut oil. This is because at a lower temperature, the coconut oil would solidify and jeopardize the manufacturing process. Microfluidization chamber failure is one of the failure modes that we cannot control, as the chamber is built in. Transfer, as a unit operation, also becomes a critical step, as a longer transfer time might lower the formulation temperature. As this process is very temperature- and time-sensitive, the initial trials were conducted to fix the process, and then the effect of formulation parameters on nanoemulsion CQAs was studied. In the formulation section, the surfactant system covers 67% of the high-risk factors due to its compatibility with oil, hydrophilic–lipophilic balance (HLB) value, and amount (Figure 2C). Varying different surfactant systems allows us to study compatibility, and varying oil-to-surfactant ratios help us to investigate the effect of oil and surfactant amount on CQAs.

### 3.4. Full Factorial Design of Experiment

**Figure 3:**
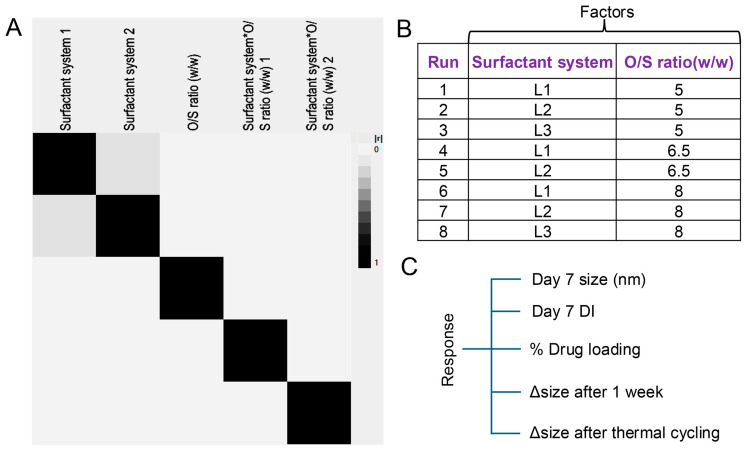
Design of experiment (DoE). (**A**) Color map on correlations among the factors showing the confounding in the experimental design. (**B**) 2 × 3 level, 2-factor full factorial design with 2 center points. (**C**) Listed responses will be checked for the 8 runs of nanoemulsion.

Surfactant system, a categorical variable with three levels (L1: 0.5% F127 + 4.5% CrEl, L2: 3% P123 + 2% F127 and L3: 0.5% F127 + 4.5% P105), and oil to surfactant ratio, a continuous variable with two levels (5 and 8), were selected to study their impact on nanoemulsion properties. Since the design comprises both categorical and continuous variables, we opted for a full factorial DoE of eight runs in total with two center points. These eight runs efficiently tested all the plausible main effects of the factors and investigated the presence of interaction among the factors. Table S2 provides the details of the DoE with the combination of the surfactant system and oil-to-surfactant ratio. Figure 3A demonstrates the presence of minimum confounding to assess the main effects and interaction effects. The independent variables are presented in Figure 3B, and the responses are listed in Figure 3C.

The responses were measured for all eight runs of the DoE nanoemulsion and analyzed based on the value specified under CQAs in Table 3. Only two runs (Run 1 and 3) met all the CQA specifications, e.g., droplet size and polydispersity index on day 7, change in size after 1 week of storage and thermal cycling, and encapsulation efficiency. The two nanoemulsions that satisfy all the CQA specifications are subjected to a stability study in serum-containing media and long-term storage stability. Based on that result, one nanoemulsion is selected for further scale-up to a 500 mL batch with and without drugs.

### 3.5. Regression Analysis and Predictive Modeling

**Figure 4:**
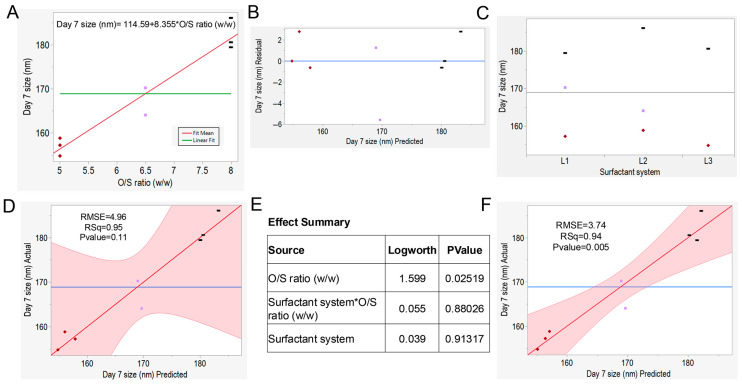
Design of experiments results: (**A**) bivariate fit of day 7 size by O/S ratio with predictive equation for day 7 droplet size; (**B**) residual vs. predicted plot of day 7 droplet size data; (**C**) one-way analysis of day 7 size by surfactant system; (**D**) actual vs. predicted day 7 size plot with interaction terms (surfactant system * O/S ratio); (**E**) effect summary; (**F**) actual vs. predicted day 7 size plot without interaction terms (surfactant system * O/S ratio).

Multiple linear regression (MLR) analysis and predictive modeling of the response based on the significant factors from the full factorial DoE were performed. The bivariate fit analysis to fit day 7 size data based on the O/S ratio is shown in Figure 4A. Quantitative bivariate fit analysis can determine the intensity of the effect of the independent variable on the response [50]. The fit line represents the predictive equation for day 7 size [Day 7 size (nm) = 114.59 + 8.36 * O/S ratio (*w*/*w*)]. In Figure 4B, the residual vs. predicted plot showed no pattern or funneling, which ensures linearity, independence, and normality in the data. Figure 4C represents the one-way analysis of the response by surfactant system, showing the distribution of day 7 size across the group. The actual vs. predicted plot of day 7 size (nm) is shown in Figure 4D. The R^2^ value of 0.95 assured the explanation of 95% variability in the prediction of day 7 size of nanoemulsion by this model. The model RMSE value was 4.96 with a *p*-value of 0.11, indicating that the model was not significant at a 95% confidence level. The effect summary table in Figure 4E lists the logWorth and *p*-value of the two parameters along with the interaction term. The O/S ratio had a significantly higher logWorth value than other factors, indicating that it has a greater impact on the model. Only the O/S ratio had a *p*-value lower than 0.05, which justified this factor as the only factor significant at a 95% confidence level. However, if the interaction term (Surfactant system * O/S ratio), which is not significant in the model, was excluded, the actual vs. predicted plot of day 7 size (nm) in Figure 4F was significant with a *p*-value of 0.005, providing statistical evidence that the observed difference between the actual and predicted values are not random but rather the impact of the factors. The low RMSE (root mean square error) value of 3.74 indicates the “good fit” of the model, suggesting closer predicted values to the actual.

### 3.6. Effect of O/S Ratio (w/w) and Surfactant System on Nanoemulsion Droplet Size

**Figure 5:**
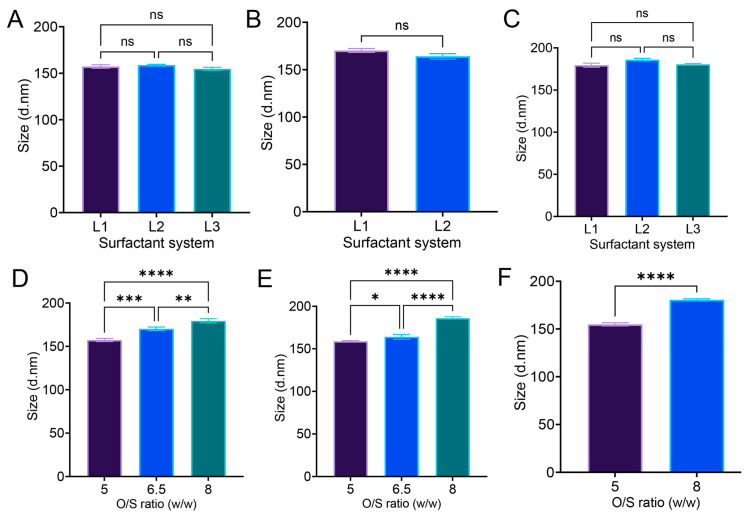
Droplet size analysis of nanoemulsion under the design of experiment. Nanoemulsion with the same O/S ratio (*w*/*w*) but different surfactant system. (**A**) O/S ratio 5, (**B**) O/S ratio 6.5, (**C**) O/S ratio 8. Nanoemulsion with the same surfactant system but different O/S ratio: (**D**) surfactant system L1, (**E**) surfactant system L2, (**F**) surfactant system L3. The data are given as mean ± SD (*n* = 3). One-way ANOVA with multiple comparison test, unpaired *t*-test, ns not significant, * *p* < 0.05, ** *p* < 0.005, *** *p* < 0.0005, **** *p* < 0.0001.

From the effect summary data in Figure 4E, the O/S ratio (*w*/*w*) appeared as the significant factor determining the droplet size of nanoemulsion, not the surfactant system. The O/S ratio is one of the driving forces for the formation of nanoemulsion, and also one of the main compositional differences between microemulsion and nanoemulsion [21]. In Figure 5A–C, no significant differences in size were observed when the O/S ratio remained the same, despite variations in the surfactant system. Three surfactant systems, L1 (0.5% F127 + 4.5% Cremophor EL), L2 (3% P123 + 2% F127), and L3 (0.5% F127 + 4.5% P105), were used in the DoE. However, in Figure 4D, nanoemulsions with the surfactant system L1 made with three different O/S ratios (5, 6.5, and 8) vary significantly in droplet diameter. For surfactant systems L2 and L3, we also notice significant differences in the droplet diameter of the nanoemulsion (Figure 5E,F). These experimental values aligned with the predictive modeling from Figure 4A; with an increasing O/S ratio, the droplet size also increases. These comparisons unequivocally tell us that, irrespective of the surfactant system, the O/S ratio (*w*/*w*) drives the generation of nanoemulsion with similar colloidal properties.

### 3.7. Colloidal and Fluorescence Stability, Morphology, and Scale-Up

**Figure 6:**
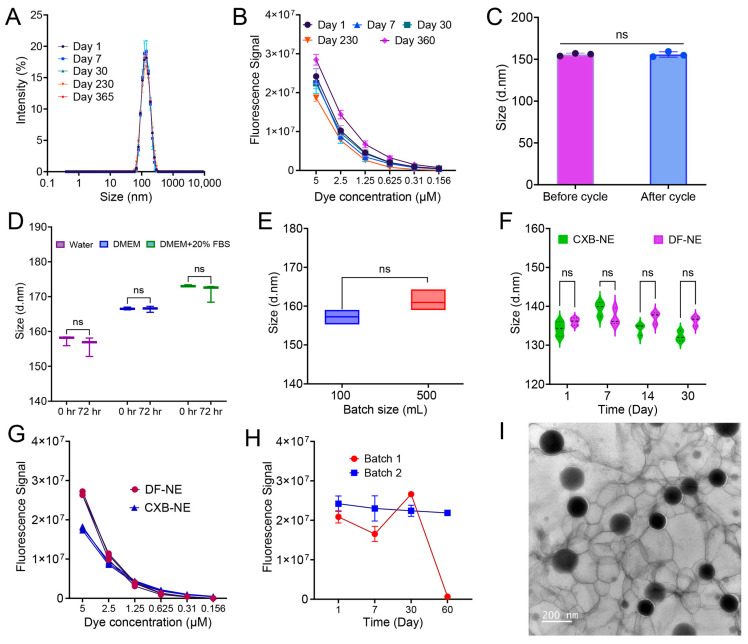
Colloidal and fluorescence stability, morphology and scale-up of DF NE and CXB NE. Twelve months storage stability in terms of the following: (**A**) size distribution; (**B**) fluorescence intensity; (**C**) stability study after thermal cycling between 4 °C and 50 °C for 8 cycles; (**D**) stability after 72 h of incubation in serum and serum-free medium; (**E**) comparison of droplet size between small-scale (100 mL) and large-scale (500 mL) batch; (**F**) comparison of droplet size between DF NE and CXB NE; (**G**) comparison of fluorescence intensity between DF NE and CXB NE; (**H**) comparison of fluorescence stability of batch 1 (without Transcutol) and batch 2 (with Transcutol); (**I**) representative transmission electron microscopic image of CXB NE. The data are given as mean ± SD (*n* = 3). Two-way ANOVA with Šídák’s multiple comparison, unpaired *t*-test, ns not significant.

To ensure the shelf life of the nanoemulsion, the nanoemulsion colloidal and fluorescence attributes need to be consistent over the storage time. The nanoemulsion droplet size and fluorescence intensity were tested on days 1, 7, 30, 230, and 365. Figure 6A,B showed the consistent droplet size distribution and fluorescence intensity of CXB NE under storage conditions. In Figure 6C, the thermal cycling stability of CXB NE results in an insignificant change in size after eight cycles of 4 °C and 50 °C. This thermal cycling study was performed to inform storage conditions and estimate the shelf-life of the nanoemulsion [43]. In Figure 6D, nanoemulsion showed insignificant size change after 72 h of incubation in water, Dulbecco’s Modified Eagle Medium (DMEM), and 20% Fetal Bovine Serum (FBS) in DMEM. This test is critical as the nanoemulsion was formulated as a parenteral theranostic platform, which would expose the nanoemulsion to biological fluid [51]. The formulation was scaled up from a 100 mL batch to a 500 mL batch. For the small-scale (100 mL) batch, we used sonication as a pre-processing step followed by microfluidization with an M110S microfluidizer. For the large-scale (500 mL) batch, we used a hand-mixer as a pre-processing unit, followed by microfluidization with an M110P microfluidizer. Although both microfluidizers have a similar type of interaction chamber and processing mechanism, they differ in terms of their processing capacity. We have successfully transferred the process from the small-scale instrument to the large-scale without having any significant differences in the colloidal properties of the nanoemulsion (Figure 6E). Figure 6F, G demonstrated that CXB NE and DF NE have no significant differences in droplet size and fluorescence intensity. This is critical to establish that CXB NE and DF NE are comparable for a logical interpretation of future in vitro and in vivo work. Figure 6H shows the importance of transcutol for the long-term stability of ICG. Batch 1 with no transcutol in the formulation showed a drastic loss of fluorescence intensity after 2 months of storage, as compared to consistent fluorescence intensity from batch 2 with transcutol. The morphological characterization from TEM showed the spherical shape of the nanoemulsion droplets (Figure 6I).

### 3.8. Celecoxib Quantification and In Vitro Release Study

**Figure 7:**
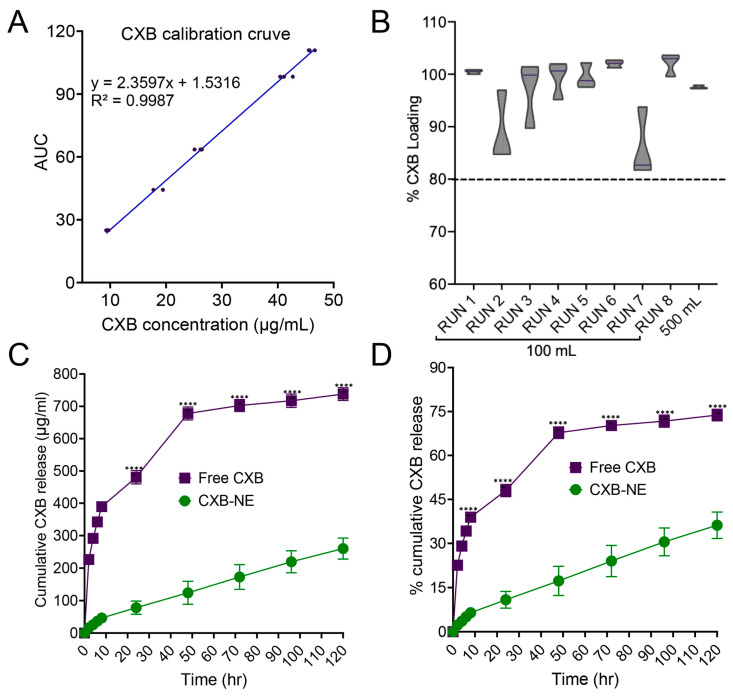
HPLC quantification and in vitro drug release study: (**A**) CXB calibration curve with triplicate data points at each concentration; (**B**) % CXB encapsulation in DoE runs, and large-scale nanoemulsion, quantified using RP-HPLC; (**C**) cumulative percentage of CXB release from nanoemulsion and free-CXB solution in release media containing 1X PBS: methanol (4:1); (**D**) cumulative amount of CXB release in µg/mL from nanoemulsion and free-CXB solution. The data are given as mean ± SD (*n* = 3). Two-way ANOVA with Šídák’s multiple comparison, **** *p* < 0.0001.

The calibration curve was developed using standard celecoxib dissolved in pure methanol. Standard concentrations from 10 μg/mL to 50 μg/mL were made by diluting in the mobile phase (75:25 methanol and water), and AUCs were plotted against the concentration to generate the curve shown in Figure 7A. The LOD (limit of detection) is 4.84 μg/mL and the LOQ (limit of quantification) is 14.67 μg/mL, calculated according to ICH Q2 (R2) [52]. A representative image of the celecoxib HPLC chromatogram is given in Figure S1. Figure 7B illustrates all the encapsulation efficiency data of the DoE batches along with the scaled-up batch. For all the batches, the encapsulation efficiency was within the range of 80–105%. An in vitro release study was performed for free CXB (celecoxib dissolved in methanol) and CXB NE in a release medium of phosphate buffered saline (PBS) and methanol (4:1). After 2 h, the cumulative CXB released from free CXB was approximately 22.60 ± 1.18%, showing a burst release compared to 2.27 ± 0.75% from CXB NE. CXB NE showed a significantly slower release of 10.87 ± 2.84% at the end of day 1, where free CXB release was approximately 48.03 ± 2.06% (Figure 7C). At the end of the experiment on day 5, approximately 300 μg/mL CXB was released from CXB NE compared to 740 μg/mL CXB from CXB solution (Figure 7D). The kinetics of the CXB release were determined based on the correlation coefficient (R^2^) value. The release kinetics from CXB NE followed the Korsmeyer–Peppas model (R^2^ = 0.99) (Figure S1). The equation followed for the calculation was:

Mt/Mα = k × t^n^

where Mt/Mα is the fraction of released drug at time t, k is the rate constant, and n is the release exponent expressing the mechanism of drug release [53]. As n = 0.71 (0.5 < n < 1), the drug release followed an anomalous non-Fickian transport mechanism. This indicated the presence of multiple dominant mechanisms along with diffusion.

### 3.9. In Vitro Cell Viability, PGE2 Release Inhibition, and Cellular Uptake Study

**Figure 8:**
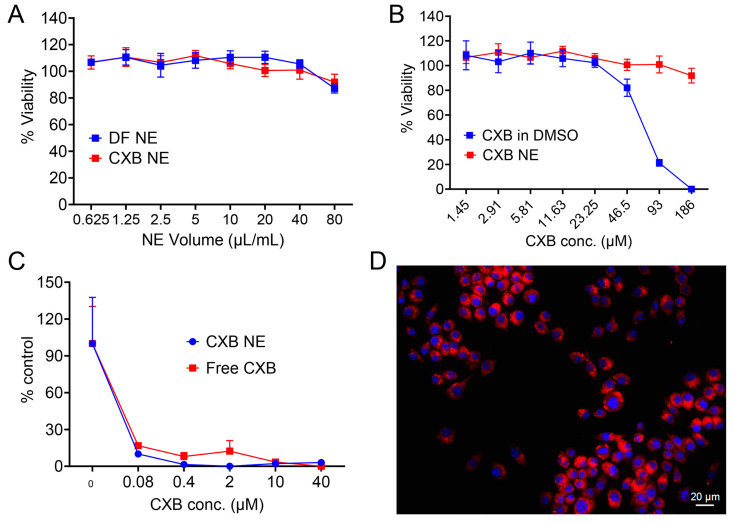
Cell culture experiments. In vitro cell viability, PGE2 release inhibition, and cellular uptake in RAW 264.7 macrophages. Macrophages were exposed to (**A**) CXB NE and DF NE. (**B**) CXB in DMSO and CXB NE for 24 h. (**A**,**B**) Assay performed using ATP-based CellTiter Glo 2.0. Data represents average ± SD, *n*= 6. (**C**) % control of PGE2 release inhibition from LPS-activated macrophages exposed to CXB NE and Free CXB at different concentrations. Data represents an average ± SD, *n* = 3. (**D**) Representative overlays of images from the DAPI (blue, nuclei) and ICG channels (purple) of RAW 264.7 macrophages treated with nanoemulsion (ICG-labeled nanoemulsion, purple). Images were taken on the Keyence microscope at 40X magnification.

Before opting for the in vitro work, the nanoemulsion cytotoxicity needed to be evaluated. An ATP-based CellTiter-Glo^®^ luminescence assay was performed to assess the viability of RAW 264.7 macrophages after 24 h of exposure to CXB NE and DF NE. The luminescence coming from the combined ATP (release from viable cells) and CellTiter-Glo^®^ was measured and normalized against the untreated group. No significant drop in ATP level was observed for either the DF NE or CXB NE groups, even at the highest concentration of 80 μL/mL nanoemulsion (Figure 8A). However, free CXB dissolved in DMSO showed a dose-dependent drop in % viability of macrophages, which is not observed at the same drug concentrations from CXB NE (Figure 8B). The toxicity of the CXB solution in DMSO was due to the drug (CXB) but not the vehicle (DMSO), as at a similar concentration, DMSO alone did not show a similar drop in macrophage viability (Figure S2). The COX-2 inhibition assay was performed using a PGE2 ELISA kit. The release of PGE2 from LPS-activated macrophages was decreased from approximately 1000 ng/mL to 150 ng/mL at a CXB concentration of 0.08 μM for both CXB NE and free CXB solution (Figure S3). The % control of PGE2, normalized based on PGE2 at no treatment, also showed a similar trend for both CXB NE and free CXB solution (Figure 8C). The uptake of CXB NE in macrophages was qualitatively tracked by using fluorescent microscopy. Brightfield microscopy was used to visualize cell morphology, while DAPI staining highlighted the cell nuclei (Figure S4). The overlay of DAPI and ICG channels indicated the presence of ICG-labeled nanoemulsion in the cytoplasm of the macrophage (Figure 8D).

## 4. Discussion

In this work, we formulated a coconut oil theranostic platform that can deliver both the payloads (drug and NIRF dye) to the activated macrophages, one of the primary markers of inflammatory pain. Coconut oil, a naturally derived biocompatible oil, shows antimicrobial [54], thermal stability [55,56] attributes, and can also act as a plasticizer [57]. Since the NIRF dye, ICG, shows thermal degradation [58], incorporation into heat-resistant solid lipid coconut oil can improve the thermal stability [37]. Moreover, different surfactant systems and the oil-to-surfactant ratio can substantially impact the colloidal properties of nanoemulsion [39,59,60]. Therefore, in this study, we have thoroughly investigated the impact of these two critical attributes by employing the QbD approach.

The presented work started by selecting the target product profile (QTPPs) for an O/W nanoemulsion with a droplet size range of 100–160 nm and a polydispersity index less than 0.2 (Table 1). A smaller droplet size with a narrow distribution ensures macrophage uptake [42,61] and adequate surface area for drug release. The critical quality attributes (CQAs) listed in Table 2 were selected to confirm colloidal stability upon storage and in the presence of biological fluid. Thermal cycling between low and high temperatures accelerates instability by increasing the rate of Ostwald ripening and altering interfacial tension [47,62]. Encapsulation efficiency > 80% and droplet size change < 20 nm after serum stability are required for the pharmacological response [7]. The initial formulations were selected based on prior lab work to investigate the pre-formulation and process parameters screening. The formulation compatibility and manufacturing feasibility were confirmed during the first trial run of a 500 mL batch. The presence of Miglyol and microfluidization at 80 and 95 psi processing pressure had no significant impact on the droplet diameter. However, sonication alone as a processing method did not achieve adequate droplet size reduction. Thus, a second processing method, microfluidization, was required. Moreover, the sonication pulse time was fixed at 30 s, as compared to a 15-s pulse time, which reduces droplet diameter significantly. However, irrespective of sonication pulse time, microfluidization lowers the droplet size in a comparable fashion, and there is no significant difference between one or two cycles of microfluidization. This led us to fix the processing parameters in both small-scale and large-scale manufacturing. Based on the insights from the pre-formulation studies, risk assessment was carried out using FMECA. The calculated RPN numbers in FMECA enabled us to categorize the failure modes systematically [10] and identify the formulation and process parameters that might have an impact on the CQAs. The process parameters with a higher RPN number were fixed based on the pre-formulation trials and prior work. This directs us to a rational approach of planning an experimental design to study the impact of the formulation parameters, which are highly likely to have an impact on nanoemulsion attributes.

A full factorial DoE was developed to study the impact of the O/S ratio (*w*/*w*) and three different surfactant systems on the colloidal properties and encapsulation efficiency of the nanoemulsions. A full factorial DoE allowed us to work with both categorical and continuous variables, along with the interaction between the variables [63]. The resulting eight runs from the DoE could be used to assess the main effects and interaction effects with minimal confounding. This confirmed the appropriateness of the design space to assess the true impact of the individual variables on the response [64]. Among the eight batches, batches 1 and 3 met all the CQA criteria. Irrespective of the surfactant system and O/S ratio, all the batches showed an encapsulation efficiency of more than 80%, which signifies the drug entrapment efficiency of coconut oil. Celecoxib was mixed only with coconut oil for an hour to dissolve completely, and we anticipated this as a probable reason for why the change in surfactant system and O/S ratio did not have a significant impact on encapsulation efficiency. However, in this study, encapsulation efficiency was not monitored over storage duration to check the formulation impact after long-term storage. The polydispersity index for all the batches was less than 0.2, implying a narrow and uniform distribution of the particles. However, with an increasing O/S ratio, the nanoemulsion droplet diameter increases, which is also evident from the bivariate fit data. An increasing O/S ratio corresponds to a decrease in surfactant concentration, which results in increased interfacial tension and thus an increase in droplet size [33,65]. The bivariate fit analysis configured the predictive equation for nanoemulsion droplet size on day 7 based on the significant factor coming from the regression analysis. The day 7 droplet size showed no trend in the one-way analysis based on the surfactant system, which explains the insignificant *p*-value in the effect summary. The regression model is not significant at a 95% confidence interval in the presence of the variable interaction term, which signifies the insignificant effect of interaction between the O/S ratio and surfactant system on nanoemulsion droplet size. Surfactant systems with varying HLB values significantly impact nanoemulsion colloidal properties [40]. However, the closer range of HLB values (13.5–16) among the three surfactant systems might be the reason for having no significant impact on the colloidal properties of the nanoemulsion. Although batches 1 and 3 met all the CQA criteria, we proceeded with batch 1 for further stability studies. This is because batch 1 was formulated with surfactant system L1, and batch 3 was formulated with surfactant system L3. The other two formulations made with L1 also survived the thermal cycling test (batches 4 and 6), which is not observed for the formulations with L3 (batches 5 and 8). Since surfactant systems influence thermal stability significantly [66], we prioritized the L1 surfactant system and opted for batch 1 nanoemulsion for further studies. The nanoemulsion retained a similar size in the presence of serum, protein, and salt-containing media, confirming the suitability of in vitro and future in vivo work [47]. The nanoemulsion was scaled up to five times the original volume using a completely different M110P microfluidizer compared to the small-scale batch (M110S microfluidizer). The comparable droplet size between the two batches signified the fact that the formulation is transferable and scalable using different manufacturing equipment. The scaled-up batch showed 1-year storage stability in terms of colloidal and fluorescence attributes, establishing formulation robustness as shown in Figure 6. To scale up the nanoemulsion to a larger scale, the adoption of Quality by Design to investigate the process parameters that have an impact on product CQAs needs to be studied. Successful scaling up to 1000 mL for a theranostic nanoemulsion has already been achieved [67]. Furthermore, the industrial microfluidizer M7250 has been used use to manufacture nanoemulsion in a 50 L batch, and a high-amplitude sonicator with liquid processor ISP-3000 achieved a manufacturing rate of 2.5 L/min [68]. Depending on the physicochemical properties, loaded drugs in nanoemulsion may localize at the aqueous phase or the oil core and affect the nanoemulsion droplet properties [69]. However, the loading of hydrophobic celecoxib in the nanoemulsion oil phase did not change the droplet size, as shown in multiple studies from our lab [12,70]. The fluorescence intensity is also comparable between the drug-free nanoemulsion (DF NE) and the celecoxib nanoemulsion (CXB NE), which is crucial for any comparative imaging of DF NE and CXB NE treatment. However, the storage stability of the NIRF dye ICG was heavily impacted in the presence of a solubilizer, transcutol. Batch 1, formulated without transcutol, retains fluorescence intensity until month 1, but drastically drops at month 2. We inferred that the stable fluorescence of ICG in batch 2 might be the result of the high solubility of transcutol, which inhibits ICG degradation in the aqueous environment. The in vitro drug release study demonstrated slower release of celecoxib from nanoemulsion compared to the celecoxib solution. The absence of the burst release of CXB from the NE scaffold reduces the chance of toxicity due to the sudden accumulation of systemic drug [71]. In vitro cell toxicity in the macrophage cell line showed no significant decrease in cell viability even at the highest exposure dose (80 μL/mL) of CXB NE and DF NE, suggesting nanoemulsion cytocompatibility. However, the free CXB in DMSO showed significant toxicity at higher concentrations. This is due to faster accumulation of CXB from the solution compared to CXB NE, which was also observed from the in vitro release study. A similar trend of decrease in PGE2 release was observed in LPS-activated macrophages treated with CXB NE and CXB solution, suggesting successful COX-2 inhibition by celecoxib-loaded nanoemulsion [27]. The highest concentration of CXB in the COX-2 inhibition study (40 μM) showed no cell toxicity in the cell viability study. Therefore, the decreased release of PGE2 results from inhibition of the COX-2 enzyme, not from macrophage cell death. This was further established by demonstrating the uptake of CXB NE in macrophages. The overlays of ICG and DAPI staining clearly showed the co-localization of the ICG nanoemulsion in the macrophage.

## 5. Conclusions and Future Perspective

In summary, we developed a coconut oil theranostic nanoemulsion encapsulating celecoxib and ICG dye with promising analgesic effects. Following a comprehensive risk assessment, we identified the critical formulation parameters that can impact the CQAs of the theranostic platform. Using a full factorial design of experiments approach, we determined that the oil-to-surfactant ratio significantly influences the nanoemulsion colloidal characteristics. Multiple linear regression and bivariate fit analysis enable statistical predictive modeling for nanoemulsion droplet diameter. The formulation was scalable with comparable attributes using different equipment, proving the flexibility of process transfer. In vitro drug release study showed no burst release of CXB, and the nanoemulsion exhibited no toxicity to the macrophage cell line. The formulation demonstrated a substantial reduction in prostaglandin E2 release with evidence of macrophage cell uptake. To our knowledge, this is the first time a celecoxib-loaded theranostic platform has been developed using plant-derived hydrocarbon oil and the QbD approach that exhibits COX-2 inhibition. This simplified formulation of nanoparticles, manufactured with only plant-derived hydrocarbon oil and pluronic surfactant systems, brings pain nanomedicine one step closer to clinical translation. Animal studies in the rodent pain model are needed to further evaluate in vivo pain relief efficacy and safety.

## Figures and Tables

**Table 1:** Quality target product profiles (QTPPs) of celecoxib nanoemulsion.

QTPPs	Target	Justification
Dosage form	O/W nanoemulsion	Enhancement of drug bioavailability
Dose	0.24 mg of CXB/kg body weight, single dose	Already proven pain relief for 4 days
Route of administration	Intravenous	Better efficacy in targeting macrophages and escaping first-pass metabolism
Encapsulation efficiency	>80%	Optimum drug encapsulation for therapeutic efficacy
Morphology	Spherical	For uniform release and high encapsulation efficiency
Droplet size	100–160 nm	Standard quality of nanoemulsion for stability
Polydispersity index	<0.20	Standard quality of nanoemulsion for stability
Stability	Stable under normal and stress conditions	Measure of normal and accelerated study

**Table 2:** Critical Quality Attributes (CQAs).

CQAs	Target	Justification
Size at day 7	100–160 nm	By day 7, nanoemulsion droplets will equilibrate to a stable size
PDI at day 7	<0.2	By day 7, nanoemulsion droplets will equilibrate, and DI will be stable
Δ size after 1 week	<10%	Gives an idea of the storage stability of nanoemulsion
Δ PDI after 1 week	<0.1	Gives an idea of the storage stability of nanoemulsion
Encapsulation efficiency	>80%	Evaluate the efficiency of the nanoemulsion to encapsulate the drug
Δ size after thermal cycling	<10%	Evaluate the shelf-life and storage conditions of nanoemulsions in harsh environments
Δ size after serum stability	<20 nm	Tested in medium and serum to assess the stability as the nanoemulsion is intended to be used intravenously, where it will encounter body fluid with electrolytes and protein.

**Table 3:** Summary of the full factorial design of experiment (DoE).

Run	Droplet Size (nm)	PDI	Δ Size After 1 Week	Encapsulation Efficiency	Δ Size After Thermal Cycling
1	157.23 ± 1.52	0.08 ± 0.01	1.02 ± 0.01%	100.47 ± 0.36%	−0.06 ± 0.01%
2	158.83 ± 0.59	0.10 ± 0.02	1.81 ± 0.01%	88.84 ± 5.75%	15.65 ± 0.01%
3	154.80 ± 1.40	0.11 ± 0.01	0.69 ± 0.01%	97.00 ± 5.19%	−1.65 ± 0.01%
4	170.23 ± 1.68	0.09 ± 0.02	0.18 ± 0.02%	99.25 ± 2.94%	9.52 ± 0.01%
5	164.06 ± 2.36	0.08 ± 0.01	−1.04 ± 0.01%	99.50 ± 1.96%	3.94 ± 0.01%
6	179.43 ± 1.98	0.08 ± 0.02	−0.09% ± 0.02%	102.00 ± 0.60%	9.97% ± 0.02%
7	186.07 ± 1.29	0.07 ± 0.02	0.61 ± 0.01%	86.05 ± 5.46%	17.48 ± 0.01%
8	180.56 ± 0.87	0.06 ± 0.03	−0.69 ± 0.01%	102.05 ± 1.79%	18.25 ± 0.02%

## Data Availability

Data can be made available for non-commercial uses and per reasonable request made to the corresponding author at janjicj@duq.edu.

## Supplementary Materials

The following supporting information can be downloaded at https://www.mdpi.com/article/10.3390/pharmaceutics17081010/s1, Table S1: Identification of the high-risk factors based on risk priority numbers (RPNs), their cause of failure, and list of CQAs that are impacted along with the source of risk. Severity, frequency, and detectability are presented as S, F, and D, respectively. Table S2: (2 × 3) level, 2-factor full factorial design of experiment with two center points. Figure S1: HPLC chromatogram and drug release kinetics model. (A) Representative image of HPLC chromatogram of celecoxib quantified from the celecoxib nanoemulsion sample; (B) drug release kinetics following the Korsmeyer–Peppas model with an R2 value of 0.99 and *n* = 0.71 following non-Fickian diffusion. Figure S2: In vitro cell viability in RAW 264.7 macrophages. Macrophages were exposed to CXB in DMSO and DMSO alone as a drug vehicle. Figure S3: In vitro PGE2 inhibition assay in RAW 264.7 macrophages. Macrophages were exposed to CXB NE and free CXB solution in DMSO. Figure S4: In vitro cell uptake study in RAW 264.7 macrophages. Representative images of RAW 264.7 macrophages treated with nanoemulsion. Images were taken with the Keyence microscope at different channels at 40X magnification (Brightfield, DAPI, and ICG).

## Abbreviations

QbD: Quality by Design; NE: nanoemulsion; PFC NE: perfluorocarbon nanoemulsion; ICG: indocyanine green; CQAs: critical quality attributes; QTPPs: quality target product profiles; DoE: design of experiment; PDI: polydispersity index; COX-2: cyclooxygenase-2; DLS: dynamic light scattering; HPLC: high performance liquid chromatography; HLB: hydrophilic–lipophilic balance.
