# Supplementary material for: Design of Experiments Leads to Scalable Analgesic Near-Infrared Fluorescent Coconut Nanoemulsions
Source: Pharmaceutics. 2025 Aug 1;17(8):1010. doi: 10.3390/pharmaceutics17081010 (PMC12388975; doi:10.3390/pharmaceutics17081010)
Supplement: Supplementary file 1 [file pharmaceutics-17-01010-s001.zip › pharmaceutics-3720753-supplementary.pdf]

SUPPLEMENTAL MATERIAL

Design of Experiments Leads to Scalable Analgesic Near-Infrared Fluorescent Coconut Nanoemulsions

Amit Chandra Das, Gayathri Aparnasai Reddy, Shekh Md. Newaj, Smith Patel, Riddhi Vichare, Lu Liu and Jelena M. Janjic *

School of Pharmacy, Graduate School of Pharmaceutical Sciences, Duquesne University, Pittsburgh, PA 15282, USA

* Correspondence: janjicj@duq.edu

Table S1: Identification of the high-risk factors based on risk priority numbers (RPNs), their cause of failure, and list of CQAs that are impacted along with the source of risk. Severity, frequency, and detectability are presented as S, F, and D, respectively.

S	F	D	RPN	CQA impacted	Risk source	Cause of failure
4	2	1	8	Day 7 size and/or PDI	Dispensing	Under and/or overweighing of materials
3	2	2	12		Magnetic stirring	Too high magnetic stirring rate
2	3	3	18		Sonication	Too low or high sonication time
5	1	2	10		Hand mixer	Decreased hand mixing time
5	3	5	75		Microfluidization	Interaction chamber failure
5	4	2	40		Amount of oil	Low or high amount of oil
4	4	2	32		Amount of surfactant	Low or high amount of surfactant
3	4	4	48	Δsize and/or PDI after 7 days	Magnetic stirring	High temperature during magnetic stirring
3	3	3	27		Microfluidization	Higher number of microfluidization passes
5	4	3	60		Amount of oil	Low or high amount of oil
3	2	2	12		Sonication	Tow low amplitude%
5	4	3	60		Amount of surfactant	Low or high amount of surfactant
5	4	3	60		Choice of surfactant	Low or high HLB
2	2	1	60	%Encapsulation efficiency	Magnetic stirring	Too low temperature
2	3	4	24		Sonication	Too high or low temperature
4	3	4	48		Microfluidization	Temperature of output emulsion
5	4	3	60		Amount of oil	Low amount of oil
4	4	4	64		Choice of surfactant	Low or high HLB

Table S2: (2×3) level, 2-factor full factorial design of experiment with two center points.

Run	Surfactant system	Oil-to-surfactant ratio (O/S ratio) (w/w)
1	0.5% F127 + 4.5% CrEl (L1)	5
2	3% P123 + 2% F127 (L2)	5
3	0.5% F127 + 4.5% P105 (L3)	5
4	0.5% F127 + 4.5% CrEl (L1)	6.5
5	3% P123 + 2% F127 (L2)	6.5
6	0.5% F127 + 4.5% CrEl (L1)	8
7	3% P123 + 2% F127 (L2)	8
8	0.5% F127 + 4.5% P105 (L3)	8

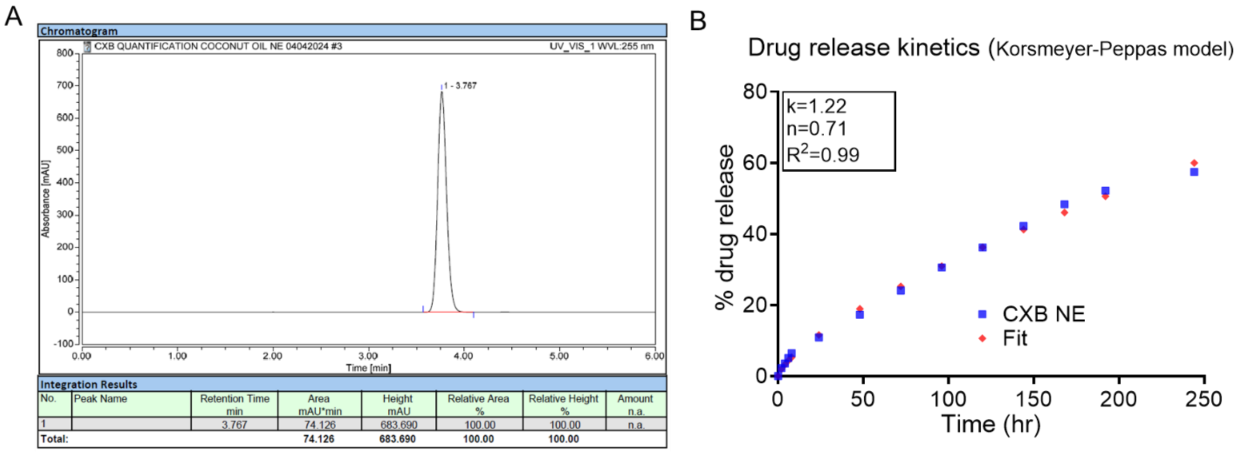

Figure S1: HPLC chromatogram and drug release kinetics model. (A) Representative image of HPLC chromatogram of celecoxib quantified from the celecoxib nanoemulsion sample and (B) drug release kinetics following the Korsmeyer-Peppas model with an R^2 value of 0.99 and n=0.71 following non-Fickian diffusion.

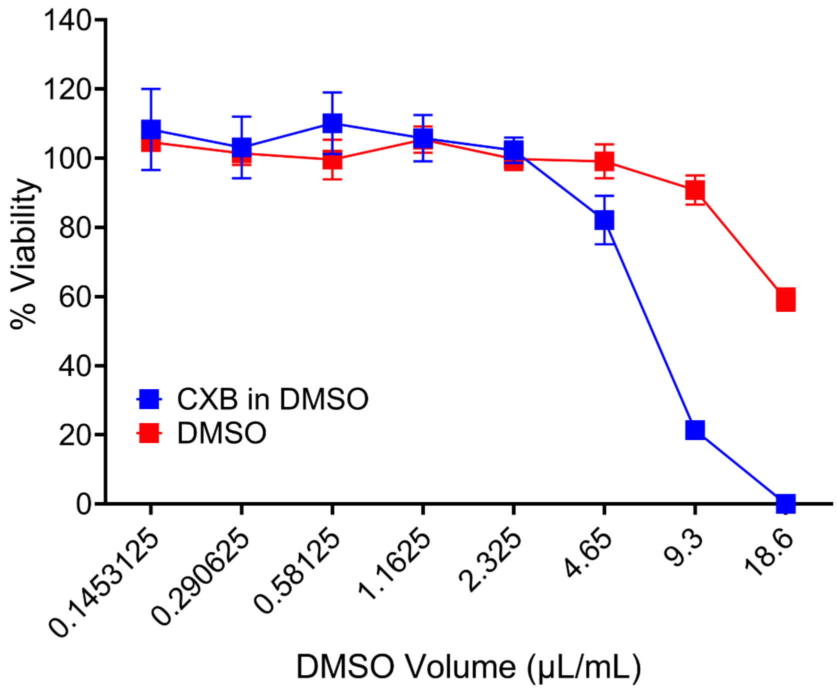

Figure S2: In vitro cell viability in RAW 264.7 macrophages. Macrophages were exposed to CXB in DMSO and DMSO alone as a drug vehicle.

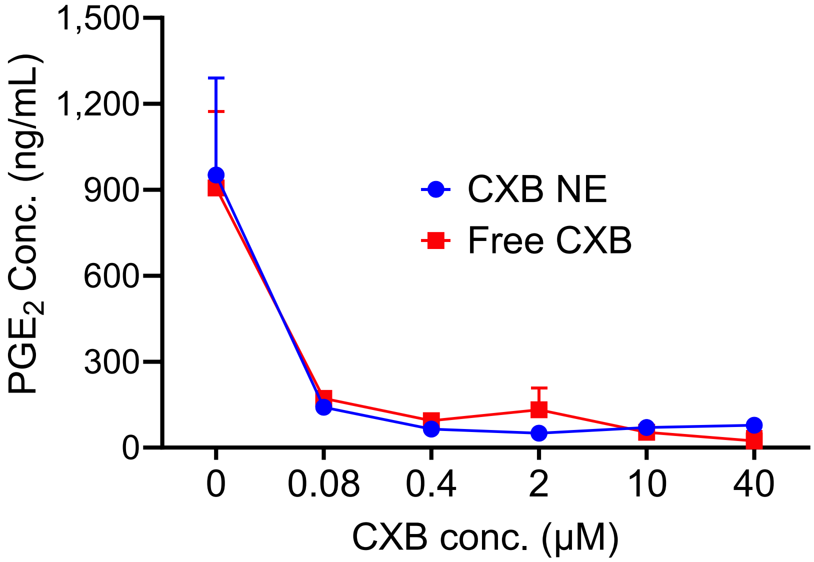

Figure S3: In vitro PGE_2 inhibition assay in RAW 264.7 macrophages. Macrophages were exposed to CXB NE and free CXB solution in DMSO.

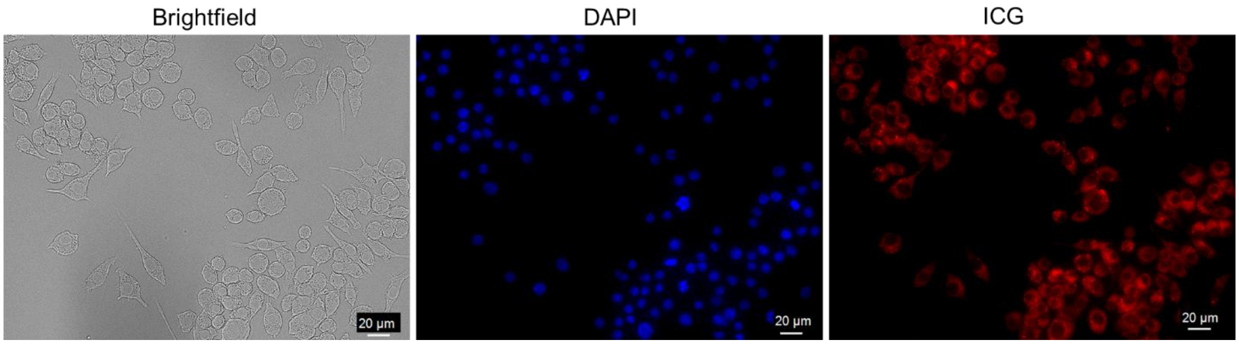

Figure S4: In vitro cell uptake study in RAW 264.7 macrophages. Representative images of RAW 264.7 macrophages treated with nanoemulsion. Images were taken with the Keyence microscope at different channels at 40X magnification (Brightfield, DAPI, and ICG).
